# What do we know about the epidemiology and the management of human echinococcosis in Albania?

**DOI:** 10.1007/s00436-023-07878-4

**Published:** 2023-06-05

**Authors:** Poleta Luga, Arben Gjata, Ilir Akshija, Ledina Mino, Valbona Gjoni, Arben Pilaca, Michael Zobi, Gabriela Equihua Martinez, Joachim Richter

**Affiliations:** 1 Institute of International Health, Global Health Center, Charité Universitätsmedizin, Corporate Member of Freie and Humboldt University Berlin and Berlin Institute of Health, 13353 Berlin, Germany; 2 Tirana/General & Digestive Surgery Department, University of Medicine, No. 3. Dibrës Str. 370, Tirana, Albania; 3 Statistics Department, University Hospital Center “Mother Teresa”, Tirana, Albania; 4 Pegasus Med Laboratory, Bulevardi “Zhan D’Ark”, Tirana, Albania; 5 Department of National Reference Laboratories, Institute of Public Health, Aleksander Moisiu Str. 80, Tirana, Albania; 6 International Hospital Tirana, Dritan Hoxha Str, Tirana, Albania

**Keywords:** Echinococcosis, *Echinococcus granulosus*, *Echinococcus multilocularis*, Albania, Zoonosis

## Abstract

Echinococcosis is a life-threatening neglected zoonotic disease. Cystic echinococcosis (CE) due to *Echinococcus (E.)* granulosus usually involves livestock and dogs; alveolar echinococcosis (AE) due to *E. multilocularis* involves rodents and canines such as foxes and dogs. Human hosts are infected accidentally via hand to mouth and/or foodborne/waterborne pathways. Albania is deemed to be endemic for cystic echinococcosis (CE), but there is a scarcity of data to confirm this. A systematic literature search was performed in PubMed, Google Scholar, and in other medical sources. Because of the scarcity of existing information, data confirming CE cases were reviewed from the medical hospital records of Albania’s largest Hospital, the Mother Teresa University Hospital (UHCMT) Tirana, and from a large private laboratory in Tirana (Pegasus laboratory). A total of eight eligible publications on 540 CE patients were found. Three hundred forty seven additional cases hospitalized in UHCMT from 2011 to 2020 were confirmed, as well as 36 laboratory cases and 10 Albanian cases notified in Germany. Taking all cases into account and considering 162 overlapping cases, 771 cases were documented from 2011 to 2020. The only case reported as AE was most likely a multi-organic CE. Surgery was the most frequent therapy approach used (84.7%). Autochthonous human CE seems to be widespread, and transmission is ongoing in Albania. CE patients in Albania undergo surgery more frequently compared with CE cases in other European countries. In order to establish a realistic estimate of prevalence and incidence of CE in Albania, mandatory notification should be reinforced. Stage-specific therapy can be used in CE to reduce therapy cost and diminish mortality by avoiding surgical overtreatment.

## Introduction

Echinococcosis, also formerly known as hydatidosis, is a widespread zoonotic parasitic infection that was described in antiquity by Hippocrates (Eckert et al. 2001). It is caused by *Echinococcus granulosus *sensu lato, comprising various species (*E. granulosus s.s.*, *E. equinus*, *E. ortleppi*, *E. canadensis*, *E. felidis*) and genotypes (G1-10) causing cystic echinococcosis (CE), as well as *E. multilocularis*, causing alveolar echinococcosis (AE), and the South-American species *E. vogeli* and *E. oligarthra* causing neotropical echinococcosis (NE) (Vuitton et al. 2020; Meléndez 2022). Echinococcosis is a neglected disease, and one million people are estimated to be infected with echinococcosis at any point in time with the loss of at least 285,000 disability-adjusted life-years (DALYs) (Budke et al. 2006; Tong et al. 2007; Brunetti et al. 2010). CE is highly epidemic in Southern Eastern Europe, Mediterranean countries, the Middle East, eastern Africa, Central Asia, Northwestern China, and in some regions of South America, with a human incidence as high as 50/100,000 persons/year (Brunetti et al. 2010; Casulli et al. 2022, 2023). *E. granulosus* eggs may be ingested by the human host after hand-to-mouth contact with contaminated matrices, such as egg-contaminated dog fur or soil, and by consumption of contaminated food or water (Tamarozzi et al. 2014). Furthermore, humans may also be infected by indirect transmission when *Echinococcus* ova are being transported from canine feces to food by flies, birds, or cockroaches. CE is classified as an “orphan disease” and mainly occurs in areas where slaughterhouse hygiene is not controlled (Hotez et al. 2012). Canines constitute the definitive host and are commonly infected through feeding with offals of infected farm animals (Brunetti et al. 2010).

Albania is a country located in south-eastern Europe partly characterized by a rural infrastructure with livestock farming (especially sheep), presence of stray dogs, uncontrolled domestic slaughter, and a high rural to urban migration rate. These factors are enabling factors for the spread of human cases to urban areas and persistence of CE. Despite the fact that this country has a seemingly high prevalence of CE, the disease is no longer considered notifiable, and Albania does not submit voluntary CE data to the European Surveillance System (ECDC 2015).

In Albania, echinococcosis was an obligatory reportable disease for the National Reporting System, but the disease has been underreported since the 1990s. CE was detected in 5 to 75% of livestock slaughtered in Albanian abattoirs, especially in sheep and cattle and to a minor extent in goats and pigs. The incidence of CE between 1958 and 1987 has been estimated as 2.05 per 100,000 inhabitants (Dizdari 1971; Melonashi 1975; Meshi and Veliu 1973; Papajani 1980).

The present study aims at investigating the current prevalence and incidence of echinococcosis in order to develop prevention strategies. Furthermore, it attempts to investigate present national therapy approaches and to explore perspectives of treatment optimization.

## Methods

A systematic PubMed and Google Scholar literature search was performed by using the main search terms “Echinococcosis” OR “Echinococcus” OR “Cystic echinococcosis” OR “Hydatid disease” OR “*Echinococcus granulosus”* OR “Alveolar echinococcosis” OR “*Echinococcus multilocularis”* AND “Albania.” Additionally, other articles from journals, international conferences, and national reports from the Institute of Public Health in Albania, which are not published in PubMed, were retrieved. Data on Albanian citizens diagnosed in Germany from 2011 to 2020 were retrieved from the Robert-Koch Institute (RKI), Berlin, Germany and were obtained through direct contact with its department of infection epidemiology. Other articles from neighboring countries of Albania regarding epidemiology and management of the disease were also taken into account.

Published sources were scarce and seemed to underreport the current situation, so it was decided to undertake a retrospective cross-sectional study between the periods of 2011 to 2020 from two different research institutions, i.e., the University Hospital Center “Mother Teresa” and Pegasus Medical Center (PMC) in Tirana. which diagnoses a high number of patients in the entire country of Albania. Approval from the Ethical Committees from the University of Tirana and the Charité Universitätsmedizin was asked for in order to allow analyzing retrospective data from the patients’ register in the University Hospital Mother Teresa and the Pegasus Laboratory Tirana (see below). Albania is a relatively small country with a high percentage of migration inside and outside the country.

Most patients with echinococcosis are diagnosed and treated in the University Hospital Center “Mother Teresa” — (UHCMT) (Albanian: *Qendra Spitalore Universitare “Nënë Tereza* — *QSUNT”*). The UHCMT is the largest medical and academic center located in the northern part of Tirana, and it is the only tertiary health center in Albania. The hospital contains nine departments with a total bed capacity of 1450 beds. Currently, UHCMT offers outpatient health service for about 300,000 people/year and hospitalizations to over 80,000 cases/year. UHCMT is a public institution and is financed by the Health.

Insurance Institute and Ministry of Health and the designated health care institution for admitting CE patients in Albania. Data were collected from the period 2011 through 2020.

PMC is a private laboratory, established in 2012 and accredited with International Organization for Standardization (ISO) with the main focus on performing medical testing, diagnosing diseases, and drug therapy monitoring. This diagnostic center includes three laboratory branches located in Tirana, offering services to patients throughout Albania. Samples are sent for echinococcosis serology to a laboratory located in Greece where an Enzyme-Linked Immunosorbent Assay (ELISA) is performed (Demeditec Diagnostics GmbH, Kiel, Germany). Data were collected between 2014 and 2020.

A patient was confirmed as suffering from echinococcosis if at least one serology test was positive and/or if imaging results were typical for echinococcosis and/or echinococcosis was confirmed through surgery and/or histopathology. Cases were included when diagnosed for the first time for echinococcosis. When patients were seen again for follow-up investigations, cases were considered duplicates, and figures were corrected to avoid overreporting. The data received were anonymized. A descriptive statistic was conducted of the data collected using Excel and the Statistical Package for the Social Sciences (SPSS) version 20.0. Strengthening the Reporting of Observational Studies in Epidemiology (STROBE) guidelines were adopted (Cuschieri 2019). The systematic review follows the Preferred Reporting Items for Systematic Reviews and Meta-Analyses (PRISMA) guidelines to ensure an accurate study (Moher et al. 2015). Annual incidences were calculated based on the national population census.

## Results

### Literature search

The PubMed literature search using the terms “*Echinococcus*” OR “echinococcosis” OR “hydatid disease” AND Albania yielded 11 hits. Single-term combinations in combination with the term “Albania” resulted in 10 hits for “echinococcosis” OR “Cystic echinococcosis,” 6 hits for “echinococcus,” 5 hits for “*Echinococcus granulosus*” or “Hydatid Disease.” Searches for “Alveolar echinococcosis” AND “Albania” yielded one hit and for “*Echinococcus multilocularis*” AND “Albania” resulted in no hits. The articles retrieved were identical to the articles, and based on the main search string, “echinococcosis” AND “Albania” and finally 10 articles in total were recorded. The Boolean Operator ‘AND’ was used to add Albanians as the population of interest (Baumann et al. 2021). Other articles from Albanian sources not published in PubMed were also considered such as 5 articles retrieved from the following sources: Journal of Microbiology and Experimentation, International Public Health Conference, Multidisciplinary Conference, Report from the National Institute of Public Health, and the Albanian Journal of Trauma and Emergency Surgery. There were in total 15 records which were screened for duplicates: Five of these records were excluded because they were conducted outside Albania and not on Albanian patients; two records have been removed as the full text was not available. After applying of the exclusion criteria, eight publications were eligible, from which five are from the PubMed search and three from other sources (Baumann et al. 2021). The only systematic publications were the works of Gjoni et al. (2008), Gjoni (2017), and of Pilaca et al. (2014), others were case records on single patients (Alimehmeti et al. 2012; Fabian et al. 2015; Maliqari et al. 2021), and one was a report only on veterinary CE (Bizhga et al. 2018). For the period of time between 2011 and 2020 and correcting for overlapping duplicates, at least 540 CE patients were found.

### Data from other sources

#### *Robert Koch Institute*, *Berlin*, *Germany*

Ten patients who had acquired echinococcosis in Albania during the period from 2011 to 2020 had been notified to the Robert Koch Institute.

#### *Admissions at University Hospital Center “Mother Teresa”*, *Tirana (UHCMT)*

Between 2011 and 2020, 401 patients were hospitalized at the UHCMT with the diagnosis of CE. Fifty two patients had been re-admitted more than once (2 to 4 times) due to relapses of CE. Figures were adjusted, and single cases were defined with a total of 347 primary cases. Three hundred twelve of 347 (89.9%) cases were admitted to surgery departments; of these, 225 (64.8%) were admitted to the general surgery department and 87 (25.1%) to a specialized surgery department. Only 35 (10.1%) of patients were admitted to non-surgical departments including gastroenterology (21/6.1%), infectious diseases (13/3.7%), and neurology (1/0.3%).

**Fig. 1:**
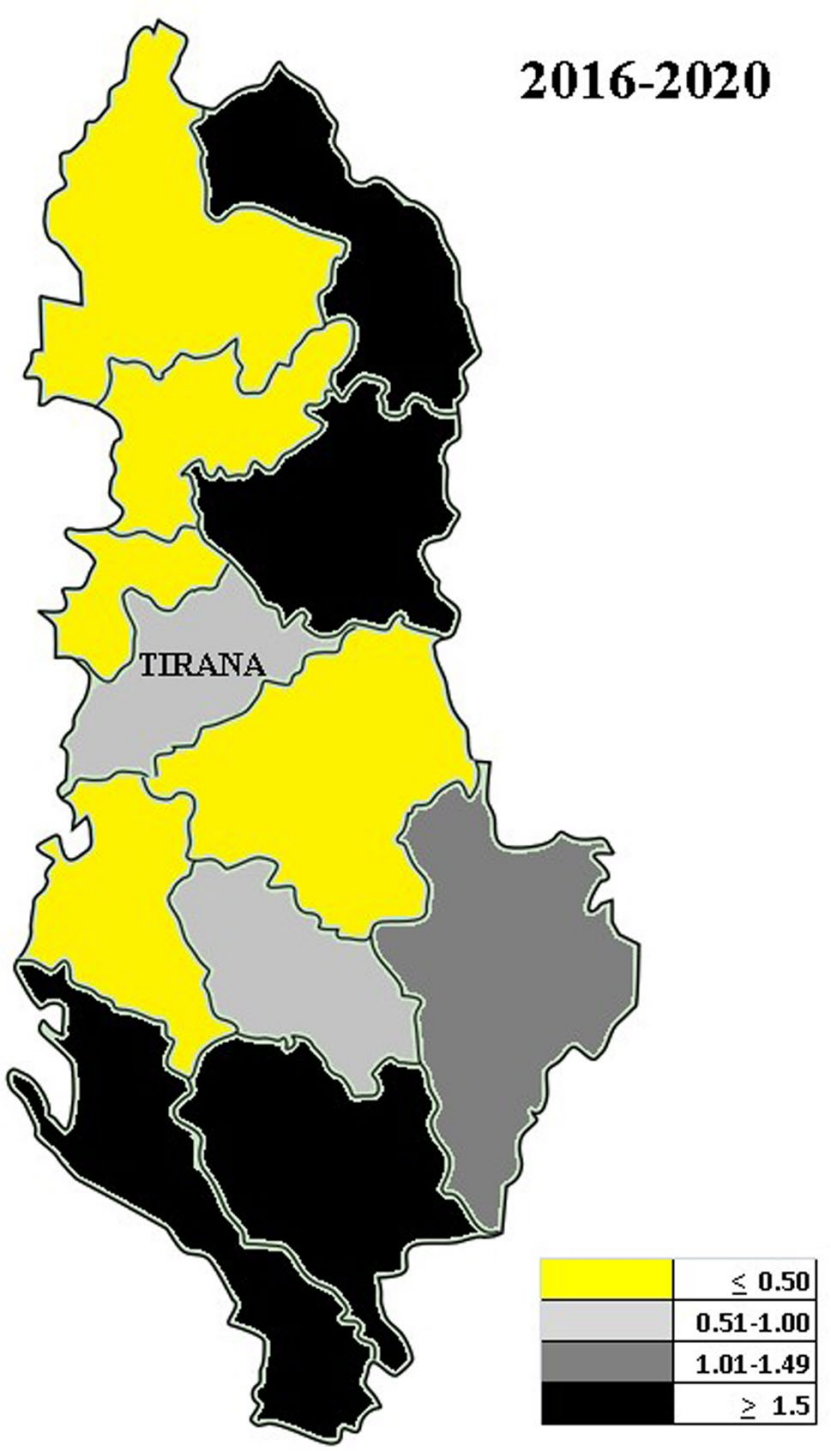
Annual incidences of cases seen at UCHMT Tirana from 2016 to 2020

The highest frequency of cases of CE within the periods between 2011 and 2020 was recorded in the year 2014. This corresponds to 13.0% (*n* = 45) of all new cases recorded in the past 10 years. This same year (i.e., 2014) had the highest number of primary cases (*n* = 45) which subsequently decreased from 2014 to 2020 (Fig. 1; Table 1). Cases of CE seen between the period of 2011 and 2020 were categorized according to geographical location with the aim to find out where the prevalence of CE is most prevalent (Table 1; Fig. 1). However, the origin of infection was difficult to locate because most cases (103/347) had been recorded as living in Tirana, the capital of Albania, where UHCMT is located and where CE was diagnosed. Considering the high migration rate of the Albanian population from rural to urban areas and the yearlong subclinical time period before echinococcosis is diagnosed, it could not always be established if infections had been acquired in Tirana or elsewhere. The district with the highest actual CE incidence was Gjirokaster followed by Kukës district and by Dibër district. Annual incidences calculated based on the annual national population census (Albanian Census of Population and Housing 2021) and estimating the minimum incidences of CE relying solely on the hospital admissions in UHCMT are presented (Table 1; Fig. 1).

**Table 1:** Annual incidences by 5-year periods and county as resulting of hospital admissions in UCHMT

District	Time period	
	2011–2015	2016–2020
Albania	1.48	0.98
Gjirokastër	3.60	3.46
Kukës	5.63	3.07
Dibër	4.38	2.46
Vlorë	1.14	1.59
Korçë	1.45	1.42
Tiranë	1.76	0.96
Berat	0.56	0.93
Durrës	0.23	0.49
Lezhë	1.49	0.47
Elbasan	0.95	0.43
Shkodër	0.28	0.29
Fier	0.52	0.20

**Fig. 2:**
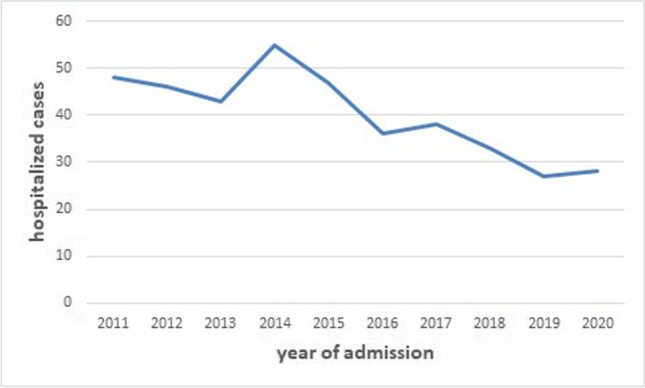
Number of CE admissions at the UCHMT Tirana

Incidences between 2016 and 2020 were lower than between 2011 and 2015 (Fig. 2). The median age of patients was 34 years; the median age of female patients (37 years) was significantly higher than that of male patients (30 years). CE was reported more frequently in female (57.3% = 199/347) than in male patients (42.7% = 148/347). Thirteen of 347 (3.7%) patients were children under 5 years confirming that transmission is still ongoing. Other 76/347 (21.9%) children and adolescents (5 to 14 years) as well as 51/347 (14.7%) adolescents and young adults (15 to 24 years) were recorded.

Cysts were most frequently located in the liver (263/347 = 75.8%), followed by the lung (25/347 = 8.1%). Nine of 347 (0.26%) cases with multiple organ involvement were recorded. Surgery rate over the years was 84.7% with no tendency of decrease over the years. The average hospitalization time was 10.3 days.

### Pegasus Medical Diagnostic Center

During the time period 2014 to March 2021 in total 36 (18.3%) of 197 tested patients had a positive serology for echinococcosis.

Taking all cases from 2011 to 2020 into account and considering further 162 overlapping cases, at least 771 CE cases were documented. Another single AE case reported in the literature was likely a misclassified multi-organic CE (Çuko et al. 2021).

## Discussion

Although Albania was known to be endemic for CE in the past, the actual prevalence and incidence is not known precisely (Dizdari 1971; Melonashi 1975; Meshi and Veliu 1973; Papajani 1980). Shiroka and Dervishi had reported a high prevalence of CE for the period of 1935–1949 of 1/3344 as reported by Gjoni and Akshija (2021). CE had been diagnosed in 1141 human cases between 1959 and 1983 (Anastasi et al. 1987; Bakalli et al. 1987), and the yearly incidence of CE between 1958 and 1987 had been estimated to be 2.05 per 100,000 inhabitants (Zanaj and Elezi 1998). Gjoni et al. reported 187 cases of CE observed in the UHCMT in Tirana from 1997 to 2006 (Gjoni et al. 2008). A study focusing on veterinary CE during a time period between 1991 and 2015 showed that CE affected not only sheep (average 42.3%), but also cattle (14.4%) and goats (5.5%) (Bizhga et al. 2018). Molecular analysis of samples confirmed *E. granulosus* genotype G1 and G3 as the causative helminth (Xhaxhiu et al. 2011; Casulli et al. 2022). CE accounted for all but one case of echinococcosis published in Albania. A similar situation has been reported from neighboring Bosnia Herzegowina (Obradovic et al. 2023). The only Albanian AE case reported in the literature was, in our opinion, a misclassified CE with multiple organ involvement to conclude from the clinical presentation and morphology of lesions described (Çuko et al. 2021). The misconception is probably also due to non-familiarity with current nomenclature (Vuitton et al. 2020). Furthermore, there was no molecular or biological proof of *E. multilocularis* as a causal agent. In a review of the global distribution of echinococcosis, AE was also not yet reported from Albania (Deplazes et al. 2017). Single cases of veterinary and/or human AE cases have, however, been reported from neighboring countries including Italy, Bosnia Herzegovina, Croatia, Serbia, and Turkey (Baumann et al. 2019; Beck et al. 2018; Deplazes et al. 2017; Ito 2017; Massolo et al. 2018; Dušek et al. 2020; Miljević et al. 2021; Omeragić et al. 2022).

Pilaca et al. reported 333 CE patients hospitalized at UHCMT between 2005 and 2011 (Pilaca et al. 2014). Gjoni analyzed a period between 2009 and 2013 and retrieved 207 CE patients hospitalized at the UHCMT diagnosed by laboratory, radiological, and surgical findings (Gjoni 2017). The same study identified that the most affected population were farmers and pet owners and particularly the ones who reported high dog contacts (Gjoni 2017). Taken together, all cases and adjusted for 162 possibly overlapping cases, a minimum of 771 cases are documented from 2011 to 2020. These figures most probably underestimate the real number of cases in Albania considering that cases may have been missed by false negative serologic results in patients who did not undergo imaging investigations (Orhun et al. 2012). During the writing of the present study, a European-wide study on the prevalence of CE has been published (Casulli et al. 2023). Albanian hospital records presented here are also mentioned in this study, but not the data of the Pegasus laboratory has not been included confirming the underreporting of CE by hospital data alone. Furthermore, the present study reveals more detailed epidemiological and clinical data which are helpful for further steps in the future control and management of CE in Albania. Through the experience of other studies, it results that hospital records largely underreport CE cases as compared to population-based surveys (Budke et al. 2006; Orhun et al. 2012; Richter et al. 2019; Casulli et al. 2023). Anyhow, our data reveal that there is a decrease of cases over the last 10 years which is likely to be due to the high emigration rate from rural areas and urbanization of the Albanian population. In the view of the long time elapsing between infection and its diagnosis which may last more than 10 years, control efforts should focus first on the place where patients have grown up rather than their actual place of living. Rural areas with most intensive sheep raising are likely to be the places of most intensive transmission such as the rural areas of Gjirokastër, Kukës, and Dibër counties.

Surgery rate was between 84 and 91%, a very high rate in comparison with other endemic countries (Pilaca et al. 2014; Gjoni 2017). Although there is no unique gold standard therapy for complex clinical cases such as CE, the high surgery rate suggests that other therapy options are rarely considered. In comparison, other European studies reported a surgery rate between 43.1 and 69.7% (Orhun et al. 2012; Velasco-Tirado et al. 2018). Surgery as the only therapy modality for CE was postulated almost a hundred years ago: Lehmann in 1928 postulated this view in the light of dissemination and allergic shock when cysts were punctured (Eckert et al. 2001). Meanwhile, since the advent of anti-helminthic drugs including benzimidazoles and praziquantel, more conservative or minimally invasive therapy options under anthelminthic coverage have replaced surgery in a significant percentage of patients (WHO-IWGE 2001; WHO 2013, 2016; Eckert et al. 2001; Brunetti et al. 2010; Neumayr et al. 2011; Richter et al. 2021). Moreover, current classifications of cysts according to cyst activity permit an expectant “watch and wait” approach for inactive cysts (WHO class CE4 and CE5) (WHO-IWGE 2001; WHO 2013, 2016; Brunetti et al. 2010; Tamarozzi et al. 2014). Early detection of a silent disease such as CE by screening high-risk populations enables the reduction of surgery rate because of the possibility of small cysts to be treated conservatively (WHO-IWGE 2001; WHO 2013, 2016; Brunetti et al. 2010; Tamarozzi et al. 2014; Lissandrin et al. 2018; Petrone et al. 2013; Richter et al. 2019).

In conclusion, although our data base is incomplete and most likely underestimates the number of CE cases in Albania, the data available suggest that CE is still highly endemic in Albania and that transmission is currently ongoing. Therefore, mandatory notification should be reinforced to enable the prevention of CE. Epidemiological studies should also investigate whether or not AE is also present. Surgery rate might be decreased by early case detection and by raising awareness of Albanian medical doctors of the WHO stage-specific approach including alternative therapy options to surgery including expectant, conservative, and minimally invasive treatments.

## Data Availability

Data supporting the findings of this study can be found in the database of the University Hospital Center “Mother Teresa” and Pegasus Medical Center, Tirana.
